# *Ganoderma lingzhi* culture enhance growth performance *via* improvement of antioxidant activity and gut probiotic proliferation in Sanhuang broilers

**DOI:** 10.3389/fvets.2023.1143649

**Published:** 2023-04-17

**Authors:** Xuzhou Liu, Liling Huang, Yan Shi, Xiaoguo Wang, Yanglan Luo, Shiyan Wei, Yanchun Qin, Yuwen Lu, Wenlong Zhang, Ying Ju, Yong Yan, Yuying Liao

**Affiliations:** ^1^Institute of Microbiology, Guangxi Academy of Agricultural Sciences, Nanning, China; ^2^Guangxi Veterinary Research Institute, Nanning, China; ^3^Yulin Institute of Microbiology, Yulin, China; ^4^Guangxi Crop Genetic Improvement and Biotechnology Laboratory, Guangxi Academy of Agricultural Sciences, Nanning, China

**Keywords:** *Ganoderma lingzhi* culture, Sanhuang broilers, fermented feeds, intestinal bacteria, metabolomics

## Abstract

**Introduction:**

The experiment was conducted to evaluate the effects of *Ganoderma lingzhi* culture (GLC) as a fermented feed on growth performance, serum biochemical profile, meat quality, and intestinal morphology and microbiota in Sanhuang broilers. In addition, the association between gut bacteria and metabolites was investigated *via* untargeted metabolomic analysis.

**Methods:**

A total of 192 Sanhuang broilers (112 days old) with an initial body weight of 1.62 ± 0.19 kg were randomly allocated to four treatments, six replicate pens per treatment with 8 broilers per pen. The four treatments contain a control diet (corn-soybean meal basal diet, CON), a positive control diet (basal diet + 75 mg/kg chlortetracycline, PCON), and the experimental diets supplemented with 1.5 and 3% of GLC, respectively. The trial includes phase 1 (day 1–28) and phase 2 (day 29–56).

**Results:**

The results showed that broilers in PCON and GLC-added treatments showed a lower FCR (*P* < 0.05) in phase 2 and overall period and a higher ADG (*P* < 0.05) in phase 2. On day 56, the concentrations of serum SOD (*P* < 0.05), and HDL (*P* < 0.05) and cecal SCFA contents (*P* < 0.05) were increased in broilers fed GLC diets. Broilers fed GLC also showed a higher microbiota diversity and an elevated abundance of SCFA-related bacteria in the caecum. The association between intestinal bacteria and metabolites was investigated *via* correlation analysis. The differential metabolites in the caecum, such as L-beta-aspartyl-L-aspartic acid and nicotinamide riboside, were identified.

**Conclusion:**

In summary, dietary GCL supplementation could increase growth performance to some extent. Moreover, GLC might benefit broilers' health by improving serum HDL content, antioxidant status, SCFAs contents, bacterial diversity, and probiotic proliferation in the caecum.

## Introduction

Animal-source foods are central to maintaining global food security, contributing 25% of protein and 18% of calorie consumption worldwide. A rapid-growing population has led to projected increases of 48 and 57% for milk and meat, respectively, in global demand between 2005 and 2050. With the increasing demand for meat products, the livestock feedstuff shortage is inevitable, as the expected expansion of feed supply will be required to improve from 6.0 to 7.3 billion (1). In commercial poultry, feed costs contribute ~70% of the total production cost. One possible way to reduce the cost is to use unconventional or alternative feed ingredients in the poultry industry. Previous studies have revealed that fermentation treatment could decrease crude fiber content but increase crude protein content in unconventional feed ingredients (2). In terms of cost efficiencies, replacing expensive ingredients such as corn might encourage the utilization of cheaper fermented unconventional feedstuffs in animal diets (3). Studies have shown increasing interest in supplementing unconventional fermented ingredients to take advantage of their positive effect, especially on production parameters and intestinal health (2, 4). It could effectively alleviate feedstuff shortage by developing new feed resources or increasing the utilization rate of conventional feed ingredients.

*Ganoderma lingzhi* (GL), also named *G. lucidum*, has been collected, cultivated, and utilized as a traditional edible-medicinal fungus in Asian countries for centuries (5). The primary bioactive substances in GL were polysaccharides, triterpenoids, alkaloids, sterols, peptides, amino acids, and mineral elements, among which polysaccharides and triterpenoids were identified as the most effective ingredients (6). Researches demonstrated that GL showed strong bioactivities, such as antioxidation, immunomodulation, anticancer action, and hepatoprotection (7, 8).

The *Ganoderma lingzhi* culture (GLC) is produced *via* solid-state fermentation with GL strain. It possesses abundant nutrients and bioactive substances, such as protein, polysaccharides, and triterpenoids. We hypothesized that GLC could be an effective feed ingredient that may positively affect the growth and health of broilers. Therefore, the study aimed to evaluate the effects of GLC addition on growth performance, serum biochemical parameters, intestinal function (morphology, microbiota, and metabolite), and meat quality in Sanhuang broilers.

## Materials and methods

### GLC production, animals, experimental design, and sample collection

A sterilized culture media (mainly wheat bran) was inoculated with GL strain and proceeded *via* solid-state fermentation for 35 days to produce GLC. Subsequently, the fermented mixed culture containing 60% moisture was dried at 60°C. The analyzed nutrient of GLC is listed in Table 1.

**Table 1:** Analyzed chemical compositions of *Ganoderma lingzhi* culture (GLC; %).

**Item**	**GLC**
Dry matter	88.26
Gross energy, MJ/kg	15.23
Crude protein	23.25
Ether extract	3.30
Ash	9.00
Neutral detergent fiber	45.00
Acid detergent fiber	13.90
Total triterpenoids	1.04
Crude polysaccharide	4.37

All values are analyzed in duplicate.

A total of 192 16-week-old Sanhuang broilers with an initial body weight of 1.62 ± 0.19 kg were purchased from a local commercial market. All the experimental birds were randomly assigned to four treatments containing six replicates of eight birds per replicate. Four treatment diets included the control diet (corn-soybean meal basal diet), the positive control diet (basal diet + 75 mg/kg chlortetracycline), and the control diet supplemented with 1.5 and 3% GLC, respectively. The composition of experimental diets is presented in Table 2. All nutrient levels met or exceeded the requirements of the yellow-feathered broiler recommended by the Ministry of Agriculture of China (NY/T 3645-2020). The trial lasted for 8 weeks which were divided into two periods. The starter period was from 1 to 28 days, and the finisher period was from 29 to 56 days of age. All broilers were raised in wire-floored cages in an environmentally controlled room with continuous light (10–20 lux). The ambient temperature was maintained at 28–32°C, and the relative humidity was maintained at 65–75%. Indoor ventilation was controlled by industrial fans. Mashed diets and water were provided *ad libitum*.

**Table 2:** Composition and nutrient levels of the experimental diets (%, as-fed basis).

**Item**	**CON/PCON**	**1.5% GLC**	**3% GLC**
**Ingredients**			
Corn	64.18	62.07	59.93
Soybean meal	30.00	30.00	30.00
Soybean oil	2.92	3.53	4.17
GLC^a^	0.00	1.50	3.00
Calcium hydrophosphate	0.10	0.10	0.10
Limestone	1.45	1.45	1.45
Salt	0.30	0.30	0.30
DL-Methionine	0.05	0.05	0.05
Vitamin–mineral premix^b^	1.00	1.00	1.00
Total	100.0	100.0	100.0
**Calculated nutrient composition**			
ME (kcal/kg)	3,063	3,063	3,063
Crude protein	18.70	18.87	19.04
Methionine	0.35	0.35	0.34
Lysine	0.97	0.97	0.96
Calcium	0.64	0.64	0.64
Available phosphorus	0.15	0.15	0.15
**Analyzed nutrient composition**			
Dry matter	90.12	89.57	89.11
Crude protein	19.56	19.67	19.23
Ash	7.65	7.89	8.04

^a^GLC, Ganoderma lingzhi culture.

^b^Premix provided per kg of diet: vitamin A, 3,250 IU; riboflavin, 2.5 mg; cholecalciferol, 1,010 IU; vitamin B 12, 0.009 mg; thiamine, 1 mg; pyridoxine, 1.2 mg; biotin, 0.04 mg; vitamin E, 75 mg; vitamin K 3, 1 mg; folic acid, 0.5 mg; nicotinic acid, 10 mg; calcium pantothenate, 5 mg; Zn, 55.2 mg; Fe, 78 mg; Cu,7.5 mg; Se, 0.16 mg; Mn, 57.2 mg; I, 0.5 mg.

The individual body weight (BW) were weighed on day 0, 28, and 56, and the amount of feed was recorded daily to calculate the average daily gain (ADG), average daily feed intake (ADFI), and feed conversion ratio (FCR). On day 56, the 24 broilers (one bird from each pen) were selected by the BW close to the average BW in every pen. The wing vein blood samples (about 5 mL) were acquired using 10 mL vacuum blood collection tube. The serum sample was then obtained at 3,000 rpm for 15 min and stored at −20°C. These same broilers were then stunned and euthanized by immersing in CO_2_, subsequently, slaughtered for sample collection. The ileum sections (about 5 cm) and breast meat samples (about 100 g) were collected. For further morphology analysis, the ileum fragments were stored in the sterile centrifuge tube (50 mL) containing 30 mL 4% paraformaldehyde at 4°C. The breast meat samples were stored at 4°C for meat quality determination. Cecal digesta samples from each bird (one bird from each pen) were collected and kept in liquid nitrogen for further short chain fatty acid (SCFA), microbiome bioinformatics, and untargeted metabolomic analysis.

### Chemical analysis

The GLC sample was assessed for dry matter (DM), crude protein (CP), ether extract (EE), and ash (9). The concentrations of neutral detergent fiber (NDF) and acid detergent fiber (ADF) were measured using fiber analyzer equipment (2010, FOSS, Denmark). The gross energy (GE) value was determined by an oxygen bomb calorimeter (C2000, IKA, DE.). The total triterpenoid content was measured by ultraviolet spectrophotometry (2550, Shimadzu, Japan). The crude polysaccharide content was detected using the phenol-sulfuric acid method (10).

### Analysis of serum indices

The contents of superoxide dismutase (SOD), catalase (CAT), malondialdehyde (MDA), total antioxidant capacity (T-AOC), and glutathione peroxidase (GSH-Px) were measured by assay kits (Jiancheng Institute of Biological Technology, Nanjing, China) and determined using automatic biochemical analyzer (model 7170, Hitachi Corp, Tokyo, Japan). The concentrations of total triglyceride (TG), total cholesterol (TC), glucose (GLU), high-density Lipoprotein (HDL), and low-density Lipoprotein (LDL) were measured by assay kits (Jiancheng Institute of Biological Technology, Nanjing, China) according to the manufacturer's instructions and determined at the wavelength of 450 nm.

### Measurement of intestinal morphology

Intestinal morphology was measured following the method described by Abdelqader and Al-Fataftah (11). In brief, the ileum samples were cleaned by physiological saline and then embedded in paraffin wax. After staining with hematoxylin and eosin, paraffin sections were prepared. Crypt depth (CD) and villus height (VH) were measured using a light microscope with the morphometric system (Nikon Eclipse E100, Japan). Then, the villus height to crypt depth ratio (V/C) was calculated.

### Analysis of intestinal SCFAs

The cecal content was collected on day 56 and used for SCFAs concentration analysis (12). In brief, 1 g of digesta was dissolved in purified water. The samples were shaken violently and centrifuged at 12,000 rpm for 15 min. The supernatant was collected and mixed with 25% (m/v) phosphoric acid. The mixture was placed on ice for 40 min before filtering into special injection bottles for testing. The SCFAs concentrations were measured by ion chromatography (model ICS3000, Thermo Crop, Sunnyvale, USA).

### Determination of meat quality

On day 56, breast meat samples (*n* = 6) were collected to analyze meat quality, including color, pH, and shear force. Meat color was expressed as lightness (L^*^), redness (a^*^), and yellowness (b^*^) and was measured by Chroma Meter (NR, Mingao company, China). The pH values at 45 min and 24 h were determined by a pH meter (IS400, SP company, USA). The pH and meat color were measured in triplicate at three different locations. The shear force was measured following the method described by Ciobanu et al. (13). Briefly, breast meat samples were cooked at 70°C for 20 min. On each piece of meat,10 cylindrical samples (diameter 10 mm × height 10 mm) were cut along the meat fiber direction and measured using a muscle tenderness meter (MAQC-12, Mingao company, China).

### 16S rRNA sequencing and bioinformatic analysis

Total bacterial DNA from cecal contents was extracted according to the method described by instruction of the E.Z.N.A.^®^ Soil DNA Kit (Omega Bio-Tek, Norcross, GA, USA). The quality of the DNA extract was checked using 1% agarose gel electrophoresis, and the concentration of DNA was determined by spectrophotometer (NanoDrop2000, Thermo Fisher Scientific, USA).

The V3-V4 region of the 16S was amplified using bacterial primers 338F (ACTCCTACGGGAGGCAGCAG) and 806R (GGACTACHVGGGTWTCTAAT). The PCR amplification of each sample was conducted with a 20 μL reaction mixture containing 5 × FastPfu Buffer (4 μL), 2.5 mM dNTPs (2 μL), FastPfu DNA Polymerase (0.4 μL, TransGen, Beijing, China), BSA (0.2 μL), template DNA (10 ng), and each primer (0.8 μL). The procedure consisted of an initial denaturation at 95°C for 3 min, the following 30 cycles of temperature gradient (95°C for 30 s, 55°C for 30 s, and 72°C for 45 s), and finally, an extension step at 72°C for 10 min using a thermal cycler (9700, ABI GeneAmp, USA). Each sample was performed in triplicate. The triplicate amplicons were pooled, electrophoresed in an agarose gel (2%), and recovered by a DNA Gel Extraction Kit (AxyPrep, Axygen Biosciences, USA).

The purified amplicons were quantified by a Fluorometer (Quantus, Promega, USA). The sequencing libraries were constructed using combining equimolar ratios of amplicons. The resulting libraries for paired-end sequencing were determined on an Illumina MiSeq PE 300 platform by Majorbio Technology Inc (Shanghai, China). The original sequence was analyzed using FASTP (https://github.com/OpenGene/fastp, version 0.20.0) for quality control, and the FLASH (http://www.cbcb.umd.edu/software/flash, version 1.2.7) was used for stitching. The OUT clustering was performed on sequences according to 97% similarity using UPARSE (http://drive5.com/uparse/, version 7.1). The silva 16S rRNA gene database (v138) was compared by RDP classifier (http://rdp.cme.msu.edu/, version 2.11) for taxonomic annotation of OTU species. The 16S function prediction analysis was determined by PICRUSt2 (version 2.2.0). All the data were analyzed through the free online platform of majorbio cloud platform (cloud.majorbio.com).

### Untargeted caecal metabolomic analysis

Metabolites of cecal contents (50 mg) were extracted by 400 ml 80% methanol (aqueous solution) with 0.02 mg/mL L-2-chlorophenylalanin as internal standard. The mixture was treated with a high-throughput tissue crusher Wonbio-96c (Shanghai wanbo biotechnology co., LTD) at 50 Hz for 6 min at −10°C, then ultrasound was performed at 40 kHz for 30 min at 5°C. The sample was placed at −20°C for 30 min to precipitate the protein. The supernatant was then carefully transferred to the sample bottles for LC-MS/MS analysis after centrifugation at 13,000 × *g*, 4°C for 15 min. UHPLC-MS/MS analysis was performed by a UHPLC-Q Exactive system of Thermo Fisher Scientific. The chromatographic conditions were as follows: 2 μL of the sample was entered into mass spectrometry detection after being separated by HSS T3 column (100 mm × 2.1 mm i.d., 1.8 μm). The mobile phases consisted of solvent A [0.1% formic acid in water: acetonitrile (95 : 5, v/v)] and solvent B [0.1% formic acid in acetonitrile: isopropanol: water (47.5: 47.5:5, v/v)]. The separation gradient was as follows: 0–0.1 min, solvent B from 0 to 5%; 0.1–2 min, solvent B from 5 to 25%; 2–9 min, solvent B from 25 to 100%; 9–13 min, the linearity of solvent B was maintained at 100%; from 13.0 to 13.1 min, the linearity of solvent B was decreased from 100 to 0%, and from 13.1 to 16 min, the linearity of solvent B was maintained at 0%. The flow rate was 0.4 mL/min. The column temperature was kept at 40°C, and all these samples were stored at 4°C during the analysis period.

The data of mass spectrometric was collected by UHPLC-Q Exactive Mass Spectrometer equipped with an electrospray ionization source. The detection mass range was 70–1,050 m/z. Full MS resolution was 70,000, and MS/MS resolution was 17,500. The conditions were performed as follows: Capillary temperature, 320°C; heater temperature, 400°C; Aux gas flow rate, 10 arb; sheath gas flow rate, 40 arb; ion-spray voltage floating (ISVF), 3,500 V in positive mode and −2,800 V in negative mode respectively; Normalized collision energy, 20-40-60 V rolling for MS/MS.

### Statistical analysis

All the data were checked for the normality of equal variances and residuals by the UNIVERIATE of SAS (Version 9.2, SAD Inst. Inc., USA), and were subjected to ANOVA by the GLM procedure of SAS. The LSMEANS was used to separate group means with Turkey's test for adjustment. The value of *P* < 0.05 was determined as significance, and a value of 0.05 ≤ *P* < 0.10 was determined as a tendency for significance.

## Results

### Growth performance and serum biochemical indices

As shown in Table 3, GLC diets had no effect on ADG, ADFI, and FCR from day 1 to 28, compared to the control diet. Broilers in the PCON treatment showed the highest ADG among different treatments. In the finisher period, we observed that dietary GLC supplementation resulted in increased ADG (*P* < 0.05) and decreased FCR (*P* < 0.05) and showed a tendency toward a reduction of ADFI (*P* < 0.10). During the overall period, the GLC-fed broilers showed a lower FCR (*P* < 0.05) compared to those fed control diets. A decreasing trend was found on ADFI (*P* < 0.10) for broilers fed GLC diets from day 1 to 56. There was no difference between ADG and FCR to broilers in GLC and PCON treatments during the overall period.

**Table 3:** Effects of *Ganoderma lingzhi* culture (GLC) supplementation on growth performance of Sanhuang Chicken during day 1–56.

**Item**	**CON**	**PCON**	**1.5% GLC**	**3% GLC**	**SEM**	***P*-value**
**Day 1–28**						
ADG, g	55.81^b^	58.94^a^	55.38^b^	54.27^b^	0.76	< 0.01
ADFI, g	326.0	324.9	317.5	315.6	4.17	0.24
FCR	5.85	5.52	5.73	5.82	0.10	0.12
**Day 29–56**						
ADG, g	62.17^b^	66.25^a^	64.43^a, b^	63.94^a, b^	0.91	0.04
ADFI, g	367.0	363.2	350.2	349.9	4.88	0.05
FCR	5.91^a^	5.51^b^	5.44^b^	5.48^b^	0.11	0.03
**Day 1–56**						
ADG, g	58.99^b^	62.60^a^	59.90^b^	59.11^b^	0.47	< 0.01
ADFI, g	346.5	344.1	333.8	332.7	4.18	0.07
FCR	5.88^a^	5.50^b^	5.57^b^	5.63^b^	0.07	0.01

^a, b^Least squares means within a row with different superscripts differ (P < 0.05). n = 6.

ADG, average daily gain; ADFI, average daily feed intake; FCR, feed conversion rate.

As shown in Table 4, broilers fed 1.5 or 3% GLC had higher HDL (*P* < 0.05) and SOD (*P* < 0.05) concentrations in serum compared to those fed the control and positive control diets. A decreasing trend of LDL content (*P* < 0.10) was also observed after GLC addition.

**Table 4:** Effects of dietary *Ganoderma lingzhi* culture (GLC) supplementation on serum profile of Sanhuang Chicken on day 56.

**Item**	**CON**	**PCON**	**1.5% GLC**	**3% GLC**	**SEM**	***P*-value**
TC mmol/L	2.43	2.68	2.61	2.68	0.14	0.55
HDL mmol/L	0.94^b^	0.99^b^	1.54^a, b^	1.90^a^	0.23	0.03
LDL mmol/L	1.22	1.41	0.95	0.56	0.22	0.08
TG mmol/L	0.72	0.71	0.73	0.71	0.03	0.95
GLU mmol/L	12.96	13.04	12.77	12.96	0.35	0.96
GSH-Px μmol/L	36.27	34.41	39.26	38.18	3.05	0.69
SOD U/mL	40.63^b^	42.01^b^	48.88^a^	49.64^a^	2.38	0.03
CAT U/mL	1.93	2.02	1.99	1.75	0.12	0.43
T-AOC U/mL	6.47	6.64	6.90	6.76	0.54	0.95
MDA mmol/mL	1.90	1.75	1.85	1.81	0.17	0.94

^a, b^Least squares means within a row with different superscripts differ (P < 0.05). n = 6.

HDL, high density lipoprotein; LDL, low density lipoprotein; TC, total cholesterol; TG, triglyceride; GLU, glucose; GSH-Px, glutathione peroxidase; SOD, superoxide dismutase; CAT, catalase; T-AOC, total antioxidant capacity; MDA, malondialdehyde; SEM, standard error.

### Intestinal morphology and SCFAs contents

**Figure 1:**
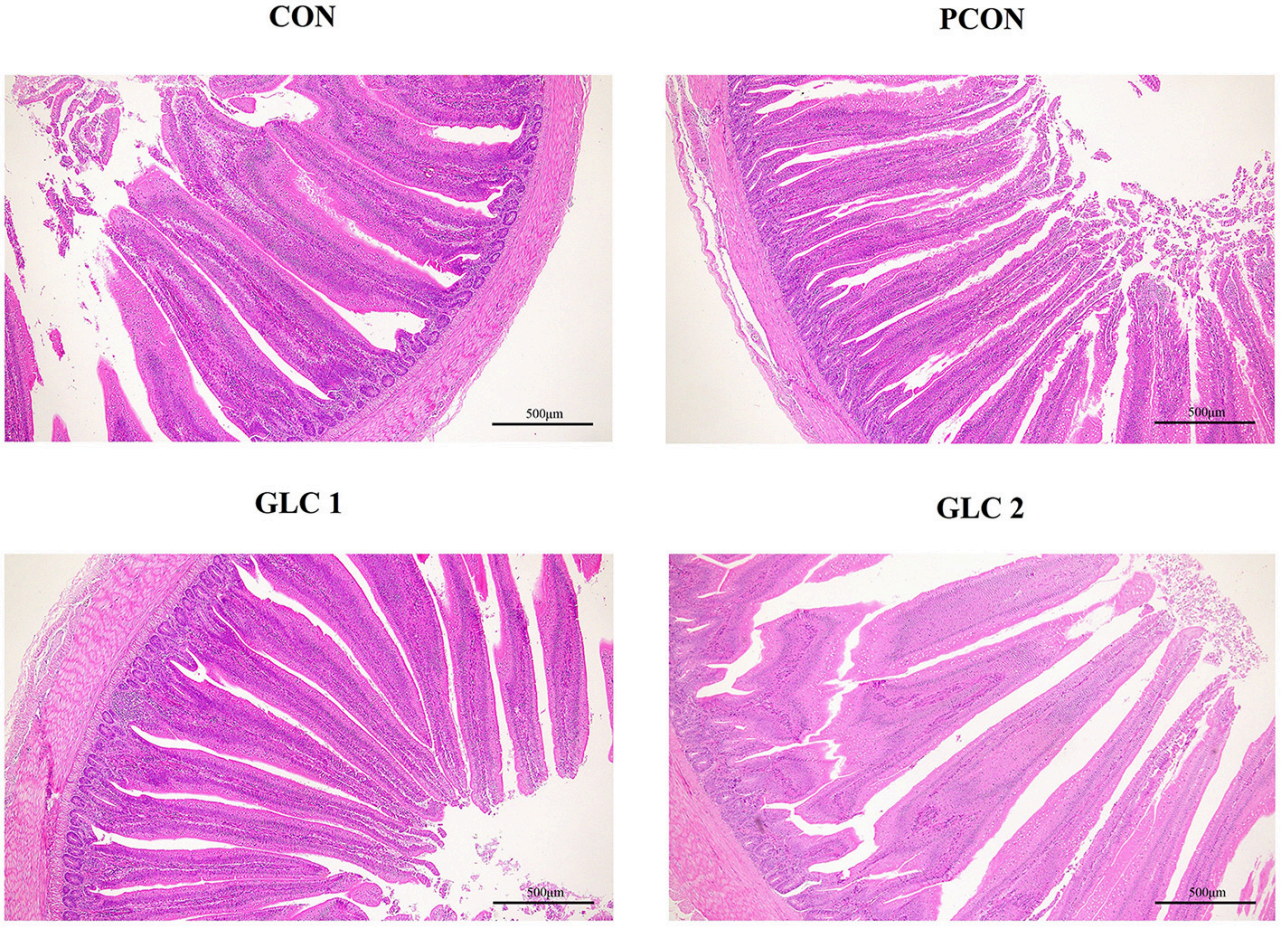
Effect of *Ganoderma lingzhi* culture (GLC) supplementation on the ileum morphology of Sanhuang broilers (*n* = 6; CON, control diet; PCON, positive control diet; GLC 1: 1.5% GCL diet; GLC 2: 3% GCL diet).

As shown in Table 5 and Figure 1, GLC supplementation showed no effect on gut morphology in broilers. Dietary GLC addition showed no effect on the acetate, propionate, or butyrate concentrations. However, increased total SCFA contents (*P* < 0.05) were revealed after GLC supplementation (Table 6).

**Table 5:** Effects of dietary *Ganoderma lingzhi* culture (GLC) supplementation on the ileum morphology of Sanhuang broilers on day 56.

**Item**	**CON**	**PCON**	**1.5% GLC**	**3% GLC**	**SEM**	***P*-value**
Villus height, μm	1,058.4	1,044.3	1,073.5	1,311.9	85.83	0.13
Crypt depth, μm	155.0	147.0	131.1	151.5	10.83	0.44
V/C	7.00	7.28	8.63	9.07	0.88	0.30

V/C, the ratio of villus height to crypt depth; SEM, standard error of mean.

**Table 6:** Effects of dietary *Ganoderma lingzhi* culture (GLC) supplementation on SCFA concentrations in cecum of Sanhuang broilers on day 56.

**Item**	**CON**	**PCON**	**1.5% GLC**	**3% GLC**	**SEM**	***P*-value**
Acetate	161.0	158.5	192.3	192.0	11.98	0.10
Propionate	84.06	85.25	92.80	91.04	4.26	0.42
Butyrate	147.4	141.2	160.3	173.3	9.92	0.15
Total	392.4^b^	385.0^b^	445.4^a^	456.3^a^	18.95	0.04

^a, b^Different superscripts within a row indicates means were different (P < 0.05).

SCFA, short chain fatty acid; SEM, standard error of mean.

### Meat quality

As shown in Table 7, GLC supplementation revealed an increased trend in pH value at 45 min (*P* < 0.10) and did not affect pH_at 24 h_, meat color, and shear force.

**Table 7:** Effects of dietary *Ganoderma lingzhi* culture (GLC) supplementation on meat quality of Sanhuang broilers.

**Item**	**CON**	**PCON**	**1.5% GLC**	**3% GLC**	**SEM**	***P*-value**
pH_at 45 min_	6.33	6.25	6.54	6.47	0.08	0.08
pH_at 24 h_	6.02	5.99	6.19	6.32	0.10	0.11
ΔpH	0.32	0.27	0.35	0.15	0.11	0.60
a${}_{\text{at }45\text{ min}}^{*}$	2.24	2.64	2.71	2.08	0.25	0.27
b${}_{\text{at }45\text{ min}}^{*}$	7.58	7.59	7.68	6.70	0.56	0.57
L${}_{\text{at }45\text{ min}}^{*}$	44.81	46.92	45.37	43.43	1.42	0.40
a${}_{\text{at }24\text{ h}}^{*}$	2.99	3.46	3.60	2.77	0.40	0.44
b${}_{\text{at }24\text{ h}}^{*}$	10.91	11.09	9.52	10.02	1.23	0.78
L${}_{\text{at }24\text{ h}}^{*}$	51.37	51.31	68.67	49.62	11.36	0.61
Shear force *N*	47.61	43.46	46.44	52.49	3.69	0.41

SEM, standard error of mean.

ΔpH = pH_at 45 min_—pH _at 24 h_; a^*^, redness; b^*^, yellowness; L^*^, lightness.

### Effect of GLC on the caecal microbiota

**Figure 2:**
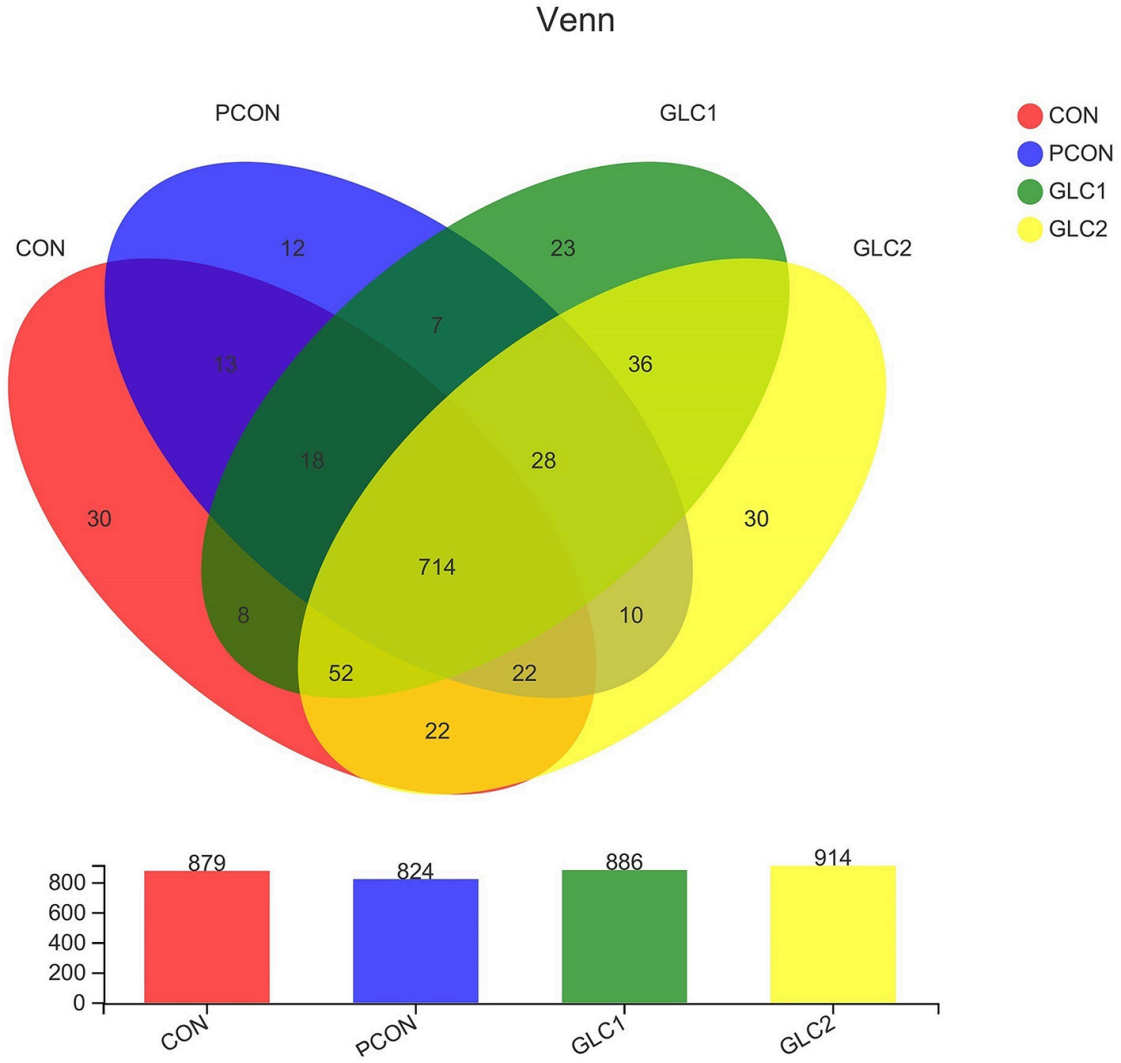
Effect of *Ganoderma lingzhi* culture (GLC) supplementation on Venn diagram analysis of the caecal microbiota (*n* = 6; CON, control diet; PCON, positive control diet; GLC 1: 1.5% GCL diet; GLC 2: 3% GCL diet).

**Figure 3:**
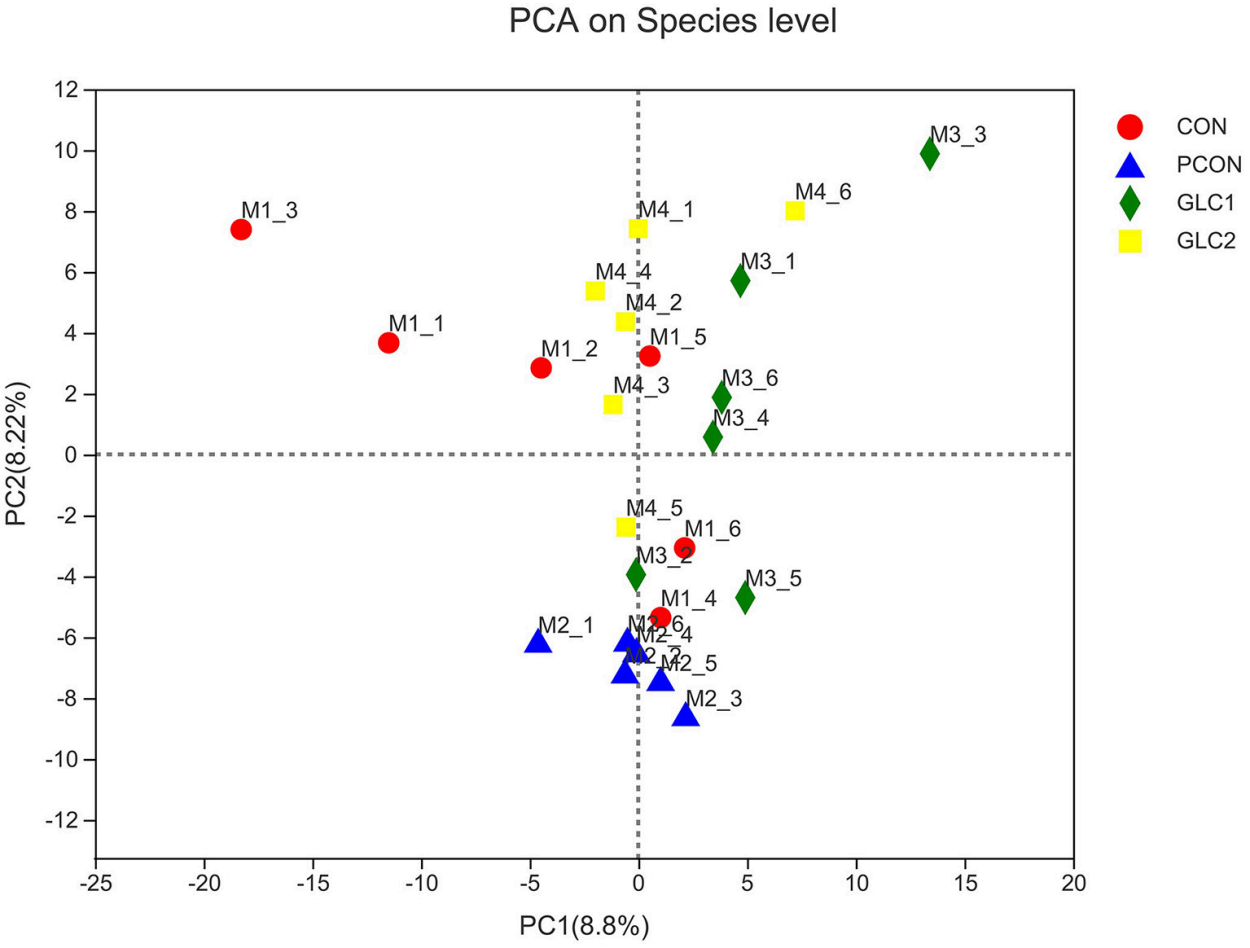
Effect of *Ganoderma lingzhi* culture (GLC) supplementation on PCA analysis of the caecal microbiota (*n* = 6; CON, control diet; PCON, positive control diet; GLC 1: 1.5% GCL diet; GLC 2: 3% GCL diet).

As shown in Figure 2, the similarity and overlap of operational taxonomic units (OTUs) among four treatments were summarized in the Venn diagram. The results revealed that all treatments shared 714 OTUs, while certain OTUs were unique to specific treatment (30 for the CON treatment, 12 for the PCON treatment, 23 for the 1.5% GLC treatment, and 30 for the 3% GLC treatment, respectively), indicating that the intervention of Chlortetracycline Hydrochloride and GLC exerted little influence on the OTUs composition of caecal bacteria in chicken. Furthermore, PCA exhibited a clear separation between CON, PCON, and GLC treatments in species-level composition (Figure 3).

**Figure 4:**
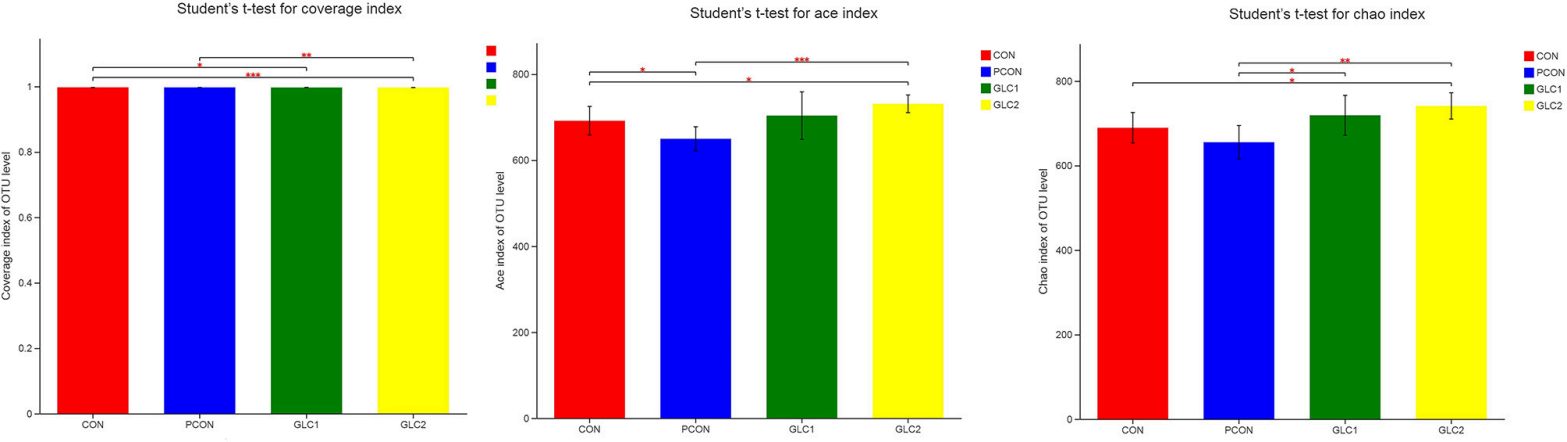
Effect of *Ganoderma lingzhi* culture (GLC) supplementation on alpha diversity of the caecal microbiota (*n* = 6; CON, control diet; PCON, positive control diet; GLC 1: 1.5% GCL diet; GLC 2: 3% GCL diet). *p < 0.05, **p < 0.01, ***p < 0.001.

**Figure 5:**
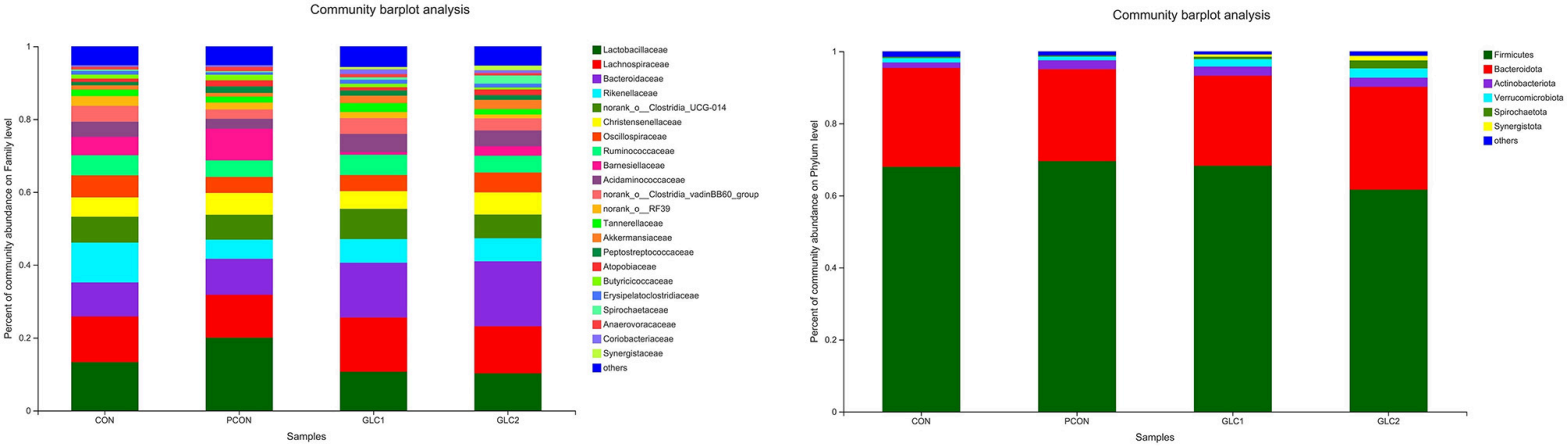
Effect of *Ganoderma lingzhi* culture (GLC) supplementation on alterations in caecal microbiota at phylum and family level (*n* = 6; CON, control diet; PCON, positive control diet; GLC 1: 1.5% GCL diet; GLC 2: 3% GCL diet).

As shown in Figure 4, the Coverage, Ace, and Chao indices of the broilers fed 3% GLC were significantly higher than those of the control and positive control treatments. The results implied that the bacterial diversity and richness of the broilers fed 3% GLC changed remarkably compared with CON and PCON treatments. As shown in Figure 5, *Firmicutes* and *Bacteroidota* hold most of the community abundance at the phylum level. Compared with CON and PCON treatment, broilers fed GLC showed a higher relative abundance of *Bacteroidaceae*, and a lower relative abundance of *Lactobacillaceae* and *Barnesiellaceae* at the family level.

**Figure 6:**
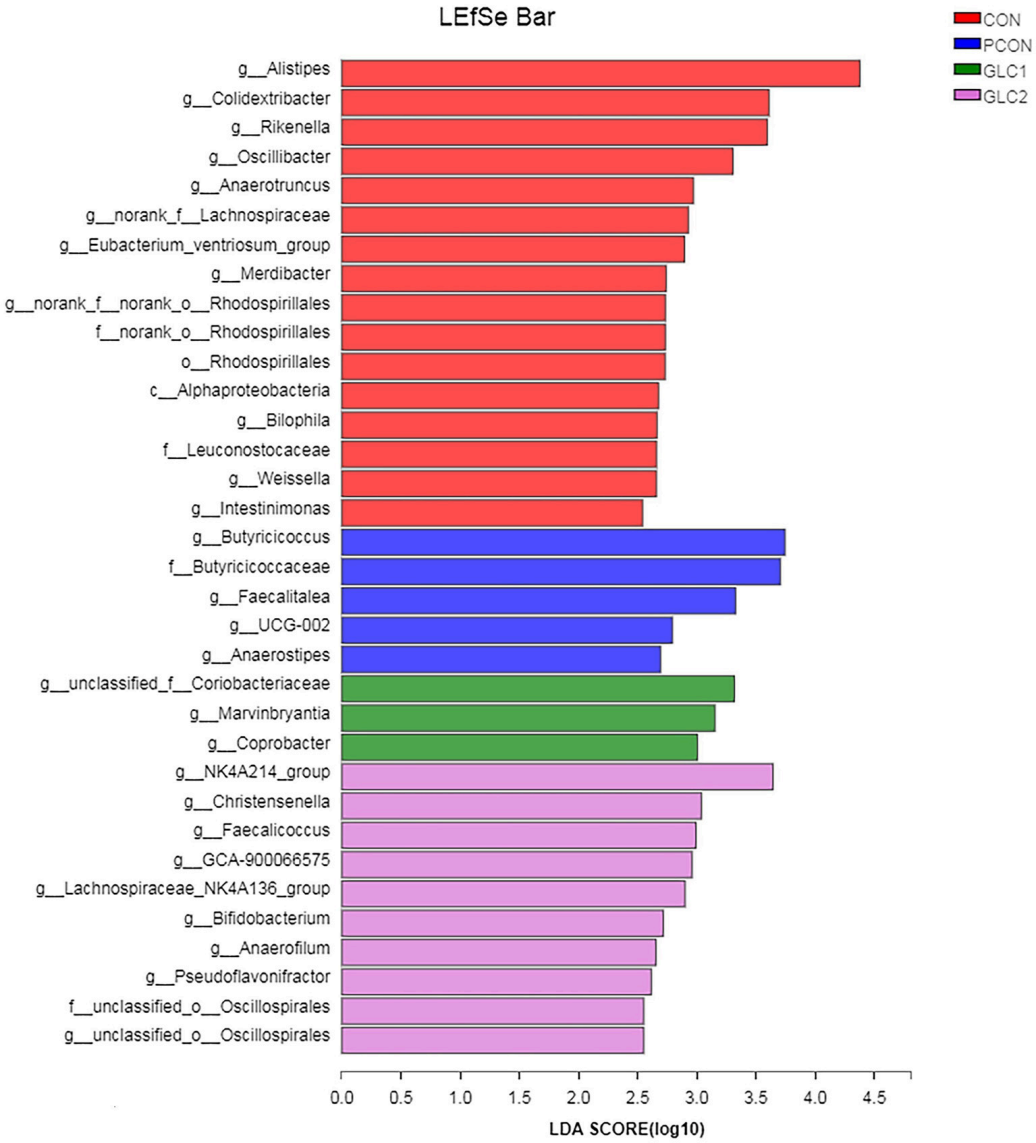
Effect of *Ganoderma lingzhi* culture (GLC) supplementation on LEfSe analysis of the caecal microbiota (*n* = 6; CON, control diet; PCON, positive control diet; GLC 1: 1.5% GCL diet; GLC 2: 3% GCL diet).

**Figure 7:**
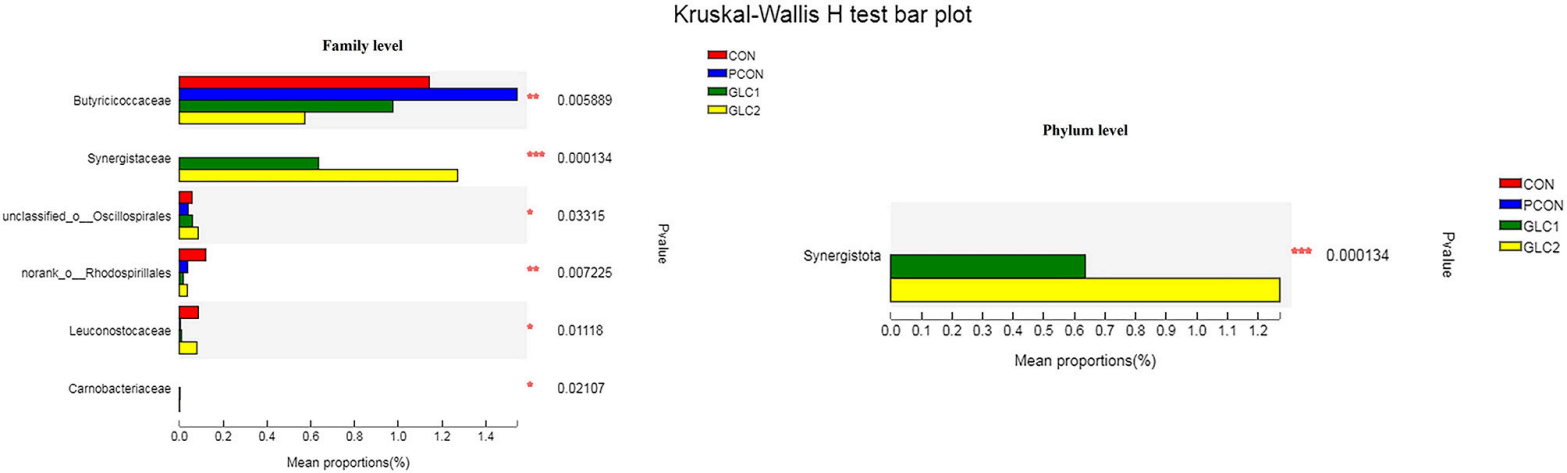
Effect of *Ganoderma lingzhi* culture (GLC) supplementation on Kruskal-Wallis *H*-test bar plot at phylum and family level of the caecal microbiota (*n* = 6; CON, control diet; PCON, positive control diet; GLC 1: 1.5% GCL diet; GLC 2: 3% GCL diet). *p < 0.05, **p < 0.01, ***p < 0.001.

As illustrated in Figure 6, the LDA score>2 means the difference was statistically significant. Compared with the CON treatment, the relative abundance of *g__unclassified_f__Coriobacteriaceae, g__Marvinbryantia, g__Coprobacter, g__NK4A214_group, g__Christensenella, g__Faecalicoccus, g__GCA-900066575, g__Lachnospiraceae_NK4A136_group, g__Bifidobacterium, g__Anaerofilum, g__Pseudoflavonifractor, f__unclassified_o__Oscillospirales*, and *g__unclassified_o__Oscillospirales* was increased significantly after GLC supplementation. In the cecum, an increase in the *Synergistota* populations on the phylum level was found in broilers fed the GLC diet (Figure 7). Moreover, on the Family level, the relative abundance of *Butyricicoccaceae* and *norank_o__Rhodospirillales* decreased while the relative abundance of *Synergistaceae* increased in the GLC treatments, compared to CON treatment.

### Untargeted metabolomic analysis of caecal digesta

#### Screening of metabolic differences

**Figure 8:**
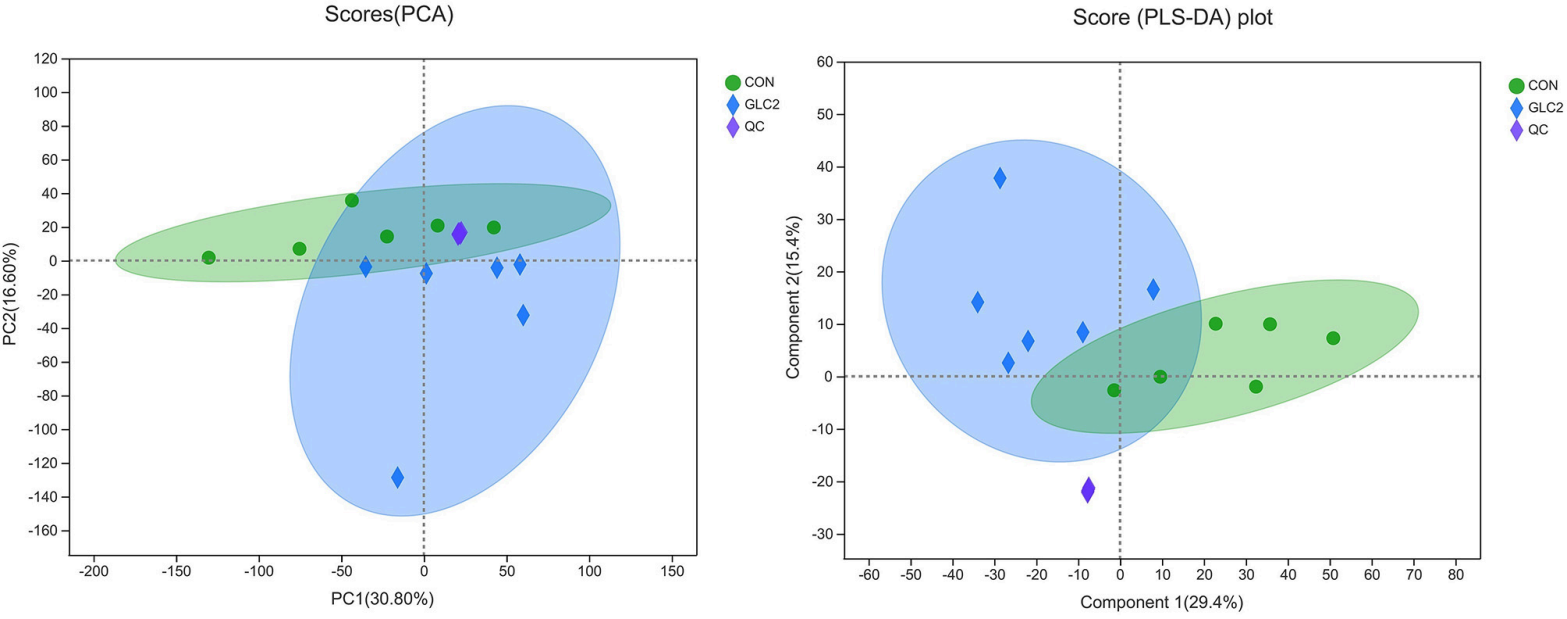
Effect of *Ganoderma lingzhi* culture (GLC) supplementation on two-dimensional PCA score of the caecal metabolites (*n* = 6; CON, control diet; GLC 2: 3% GCL diet; QC, quality control).

The results have shown that the caecal microbiota profiling of broilers changes significantly after supplementation of 3% GLC. Therefore, metabolomics was employed to ascertain the changes in microbiota metabolites, further revealing the underlying mechanism of GLC improving growth to broilers. Metabolic changes of CON and 3% GLC samples were estimated by PCA (an unsupervised principle component analysis, Figure 8). The QC samples were gathered closely, indicating that UHPLC-MS/MS system showed good stability. Meanwhile, the samples of 3% GLC treatments were not significantly separated from the CON treatment, which demonstrated that the metabolome of the samples was mostly similar. The PLS-DA was used to generate the two-dimensional score plots. The CON and 3% GLC treatments have partly overlapping distributions of data points, suggesting that the metabolome of samples in CON and 3% GLC treatments are similar.

Furthermore, the results of differential metabolite analysis between CON and 3% GLC treatments showed (VIP > 1 and *P* < 0.01) that 170 metabolites with significant differences were identified between CON and 3% GLC treatments. As shown in Supplementary Table 1, the top 50 metabolites most likely to be potential biomarkers were screened. The data have shown that, compared with CON treatment, the levels of 17 kinds of organic acids and derivatives [L-beta-aspartyl-L-aspartic acid, 2-Amino-4- [(2-hydroxy-1-oxopropyl) amino] butanoic acid, methionyl-Proline, etc.], two kinds of organic oxygen compounds (nicotinamide riboside, 5,6-Dihydrouridine), eight kinds of lipids and lipid-like molecules (3-carboxy-4-methyl-5-pentyl-2-furanpropanoic acid, menthone 1,2-glyceryl ketal, 8-Deoxy-11-hydroxy-13-chlorogrosheimin, etc.), two kinds of benzenoids (epinephrine, 5-(3′,4′-Dihydroxyphenyl)-gamma-valerolactone), three kinds of nucleotides and analogs (2′,3′-Dideoxy-3′-fluorouridine, 5-Methyldeoxycytidine, cotinine glucuronide), three kinds of organoheterocyclic compounds (pyridoxine, 2′,3′-Didehydro-2′,3′-dideoxyguanosine, gravacridonediol), two kinds of phenylpropanoids and polyketides (3-Hydroxycoumarin, liquiritigenin), one kind of alkaloids and derivative (1,2,3,4-Tetrahydro-b-carboline-1,3-dicarboxylic acid), and seven other metabolites increased. While levels of 3-alpha,20-alpha-Dihydroxy-5-beta-pregnane 3-glucuronide, 3-(3,4,5-Trimethoxyphenyl) propanoic acid, 18R-hydroxy-5Z,8Z,11Z,14Z,16E-eicosapentaenoic acid, (3S,5S)-3, 5-Diaminohexanoate, and 1 other metabolite decreased.

**Figure 9:**
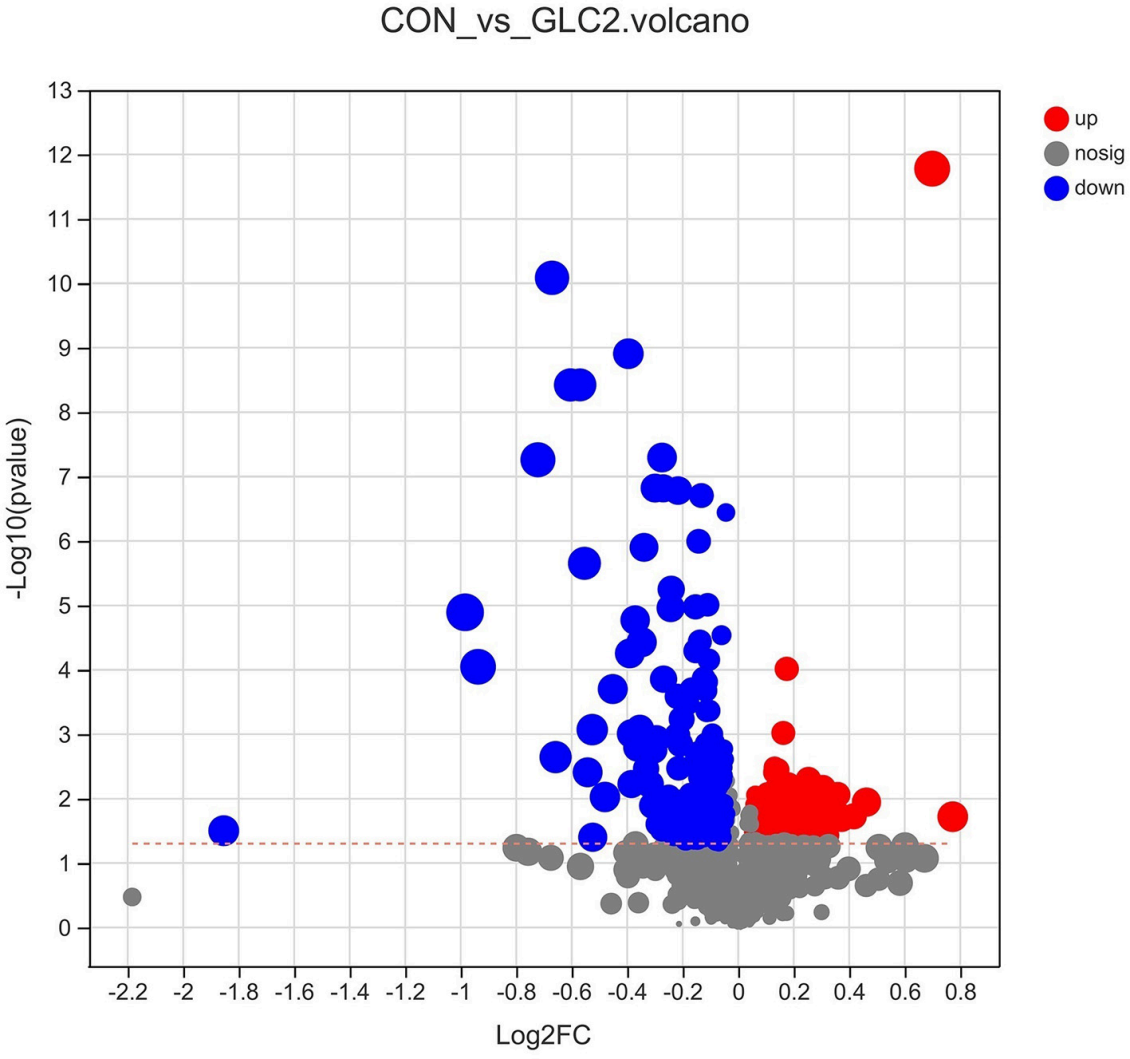
Effect of *Ganoderma lingzhi* culture (GLC) supplementation on volcano plot analysis of the caecal metabolites (*n* = 6; CON, control diet; GLC 2: 3% GCL diet).

**Figure 10:**
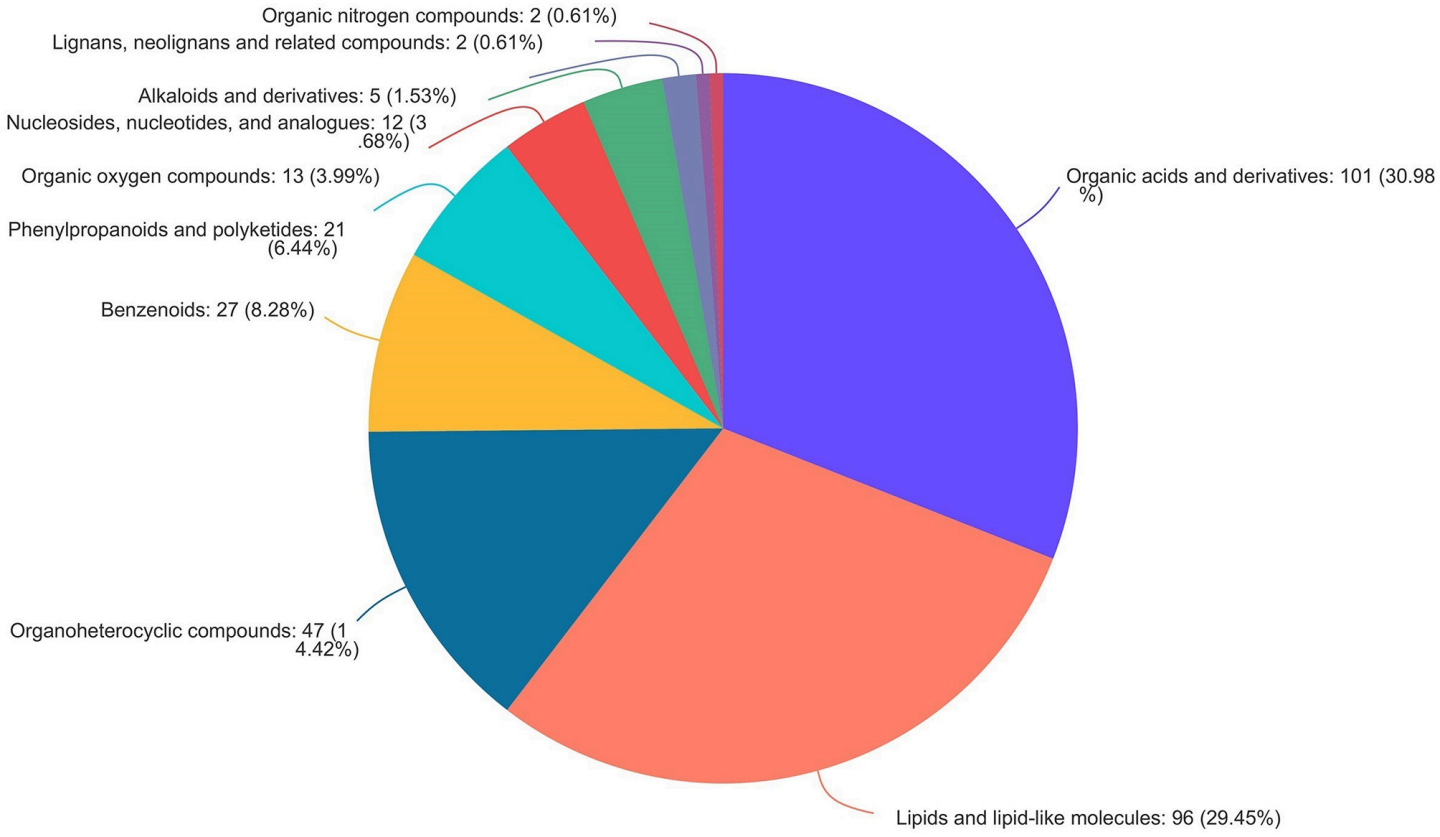
Pie chart of HMDB chemical taxonomy (super class) analysis of the caecal metabolites (*n* = 6).

As shown in Figure 9, the volcano map was constructed to visualize the potential biomarkers between CON and 3% GLC treatments. The ordinate was -log 10 (*P*-value), and the abscissa was log 2 (fold change). Each metabolite was represented by a point. The L-beta-aspartyl-L-aspartic acid, nicotinamide riboside, 2-amino-4-[(2-hydroxy-1-oxopropyl) amino] butanoic acid, methionyl-proline, topaquinone, and 8-azaadenosine were selected as biomarkers between the CON and 3% GLC treatments. All metabolites obtained from level-two identification were assigned to the HMDB (Human Urine Metabolome Database) database, 326 of which were matched and classified into 10 HMDB super classes. Meanwhile, “organic acids and derivatives,” which contained 101 metabolites, was the first class (30.98%), followed by “lipids and lipid-like molecules” (96 metabolites, 29.45%) and “organoheterocyclic compounds” (47 metabolites, 14.42%; Figure 10).

#### Correlation analysis between microbiota and metabolites

**Figure 11:**
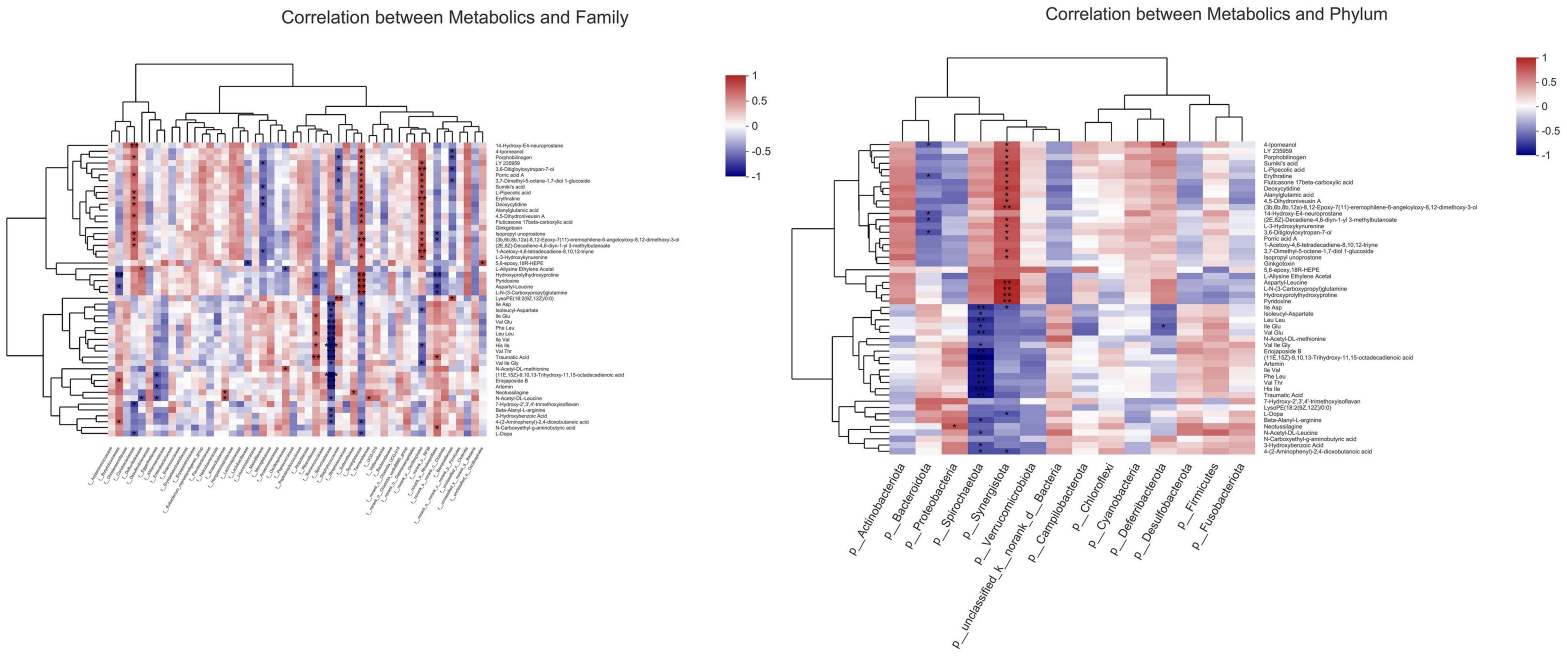
Correlation analysis between metabolites and microorganisms at phylum and family level (*n* = 6). *p < 0.05, **p < 0.01, ***p < 0.001.

As shown in Figure 11, to further investigate the potential functional connection between microorganisms and metabolites, a correlation heatmap was established according to Pearson's correlation analysis at the phylum and family level. The relative abundance of *f__Synergistaceae* was positively correlated with hydroxyprolylhydroxyproline, pyridoxine, aspartyl-leucine, and L-N-(3-Carboxypropyl) glutamine. The hydroxyprolylhydroxyproline and aspartyl-leucine were negatively correlated with the abundances of *f__Butyricicoccaceae, f__Rikenellaceae*, and *f__norank_o__Rhodospirillales*. The L-N-(3-Carboxypropyl) glutamine was positively correlated with the relative abundance of *f__Synergistaceae*, and negatively correlated with the relative abundance of *f__norank_o__Rhodospirillales*.

## Discussion

The current study detected a notable decrease in FCR of birds-fed GLC during the finisher and overall period, compared to the control diet. Similar studies have shown that the bioactive substance contained in GL could remarkably improve the growth performance of broilers. Dietary supplementation of 200 mg/kg GL spores to aflatoxin B1-contaminated diets could counteract its adverse effects on the growth performance of broilers during the overall period (6). Moreover, broilers fed diets supplemented with GL spores revealed an increased ADG and decreased FCR during the finisher period (14).

The mechanism by which GLC reduces FCR has yet to be fully elucidated. It could be partially attributed to the antioxidative characteristics of GLC. During the finisher period, growing broilers produce enhanced oxidative free radicals and endogenous calories due to body weight-correlated changes in endocrine function, body composition, and metabolic rate (14). We observed an increased antioxidant enzyme activity after GLC supplementation. The previous study showed that the GL polysaccharides could improve serum GSH-PX and SOD and reduce MDA in mice with chronic pancreatitis (15). The spores of GL might remarkably mitigate the oxidative stress level in diabetic mice and promote the recovery of body weight loss under stress (16).

We also found that broilers fed GLC showed a decreasing trend on ADFI. We deduced that the higher dietary fiber content in GLC (NDF: 45.00% and ADF 13.90%) might negatively affect the ADFI of broilers. Other studies reported that broilers fed higher fiber diets had a lower feed intake than control diets (17, 18).

We observed that GLC supplementation could increase serum HDL and show a decreased trend in LDL levels. The previous study found that GL polysaccharide complex could significantly down-regulate the low-density lipoprotein cholesterol (LDL-C) in the serum of pre-diabetic mice (19). Reactive oxygen species (ROS) and free radicals, as side-products of metabolic processes, might seriously injure cells *via* oxidation processes. We found that GLC could improve SOD levels in broilers, which is probably due to the polysaccharides and triterpenoids in GLC. Evaluating the antioxidant activity of GL polysaccharides or triterpenoids has been done *via* different methods *in vitro* or *in vivo*. All polysaccharides or triterpenoids derived from different parts of GL have confirmed the ROS scavenging abilities (20). A study showed that triterpenoids obtained from GL could improve the activities of SOD, CAT, and antioxidant enzymes in mouse splenic lymphocytes *in vitro* (21). The polysaccharides from GL have been demonstrated the capacity to induce the synthesis of SOD and CAT, scavenging on DPPH, and reducing lipid peroxidation (22). In another study, a GL polysaccharide showed a potent antioxidant effect *via* improving SOD, GSH-Px, and CAT activities in the liver of type 2 diabetic rats (23).

We found the GLC addition could promote total SCFAs content in the cecum of broilers, and the dietary fiber (especially β-glucan) in GLC might play a vital role. The dietary fiber, such as β-glucan and pectin, could not be digested directly in the upper gastrointestinal tract. The SCFAs are the primary metabolites produced by microbiota *via* fermenting dietary fiber in the large intestine. Xie et al. (24) found that the GL polysaccharides consist of β-1,3/1,6-glucan and some other carbohydrates, in which β-1,3-glucan was the main active ingredient. The GL polysaccharides consumption could produce more SCFAs (acetate, propionate, and butyrate) by increasing the SCFA-producing bacteria population, such as *Ruminococcus_1*, in the cecum of the rat. In the study of Zhu et al. (23), dietary supplementation of polysaccharides isolated from GL could significantly increase the SCFAs contents in serum, liver, and feces of diabetic rats after treatment for 4 weeks.

As is well-known, meat pH value is associated with muscle lactic acid content derived from anaerobic glycolysis (25). A higher meat pH value always correlates with decreased drip loss (higher water-holding capacity), darker color, and improved firmness. Higher ultimate pH meat would produce more acceptable quality by reducing the risk for higher drip loss and pale, soft, exudative (PSE) meat (26). Interestingly, we observed that GLC supplementation showed a trend to improve breast meat pH value at 45 min. That is, GLC addition could slow down glycolysis, thus, decreasing lactic acid concentration to some extent. However, the underlying mechanism of its protective actions remains to be explored.

The intestinal microorganisms are complex communities and have established a close symbiotic relationship with their host that contributes to the maturation of the immune system, prevention of pathogen colonization, synthesis of some vitamins, and metabolism of indigestible components (27). We assumed an underlying relation exists between the decreased FCR and higher microbiota diversity after 3% GLC addition. Previous studies suggested that higher microbial diversity is associated with more productive and stable ecological communities and stronger resistance to invasive species, leading to more efficient dietary utilization (28, 29). Zhou et al. have revealed that the intestinal microbiota is strongly linked to chicken health and phenotypes, such as feed efficiency (30).

In the current study, the relative abundance of *Firmicute* and the ratio of *Firmicutes* to *Bacteroidetes* (F/B) decreased in the 3% GLC treatment, compared to the CON treatment (2.84–2.41). Krajmalnik-Brown et al. (31) have proposed that the *Firmicutes* were more efficient in extracting energy from diets, compared to *Bacteroidetes*, resulting in higher absorption of calories and subsequent weight gain, inconsistent with our results. Interestingly, as mentioned above, the increased bacterial diversity caused by GLC addition could be linked to improved feed efficiency. We assumed that feed efficiency is affected by many factors, such as digestion and absorption in the gastrointestinal tract and microbial fermentation in the hindgut. This complexity could lead to a discrepancy. In the study of Chang et al. (32), they revealed that a water extract of *Ganoderma* mycelium (WGM) could modulate intestinal microbiota composition, mainly by decreasing the F/B ratio in mice.

In addition, the GLC supplementation could increase the relative abundance of the phylum *Synergistota*, in which the bacteria were mainly anaerobic microorganisms (33). Few studies about *Synergistota* were found, and the effect of the improved community of *Synergistota* on broilers needs further investigation. We also found that broilers fed GLC had a higher abundance of *Bacteroidaceae*. Wang et al. also observed that the β-glucan is fermented in the lower gastrointestinal tract, resulting in a microbiota composition shift, especially the improved *Bacteroidota* and reduced *Firmicutes* (34). The species of *Bacteroidaceae* are common intestinal bacteria members in all warm-blooded animals and could be considered probiotics (35).

A previous study indicated that the polysaccharides extracted from GL could increase the relative abundance of *Lachnospiraceae*, which is in accordance with our findings (36). *Lachnospiraceae*, as a beneficial bacterium, could participate in carbohydrate fermentation into gases (CO_2_ and H_2_) and SCFAs (37). The suppression of fermentation-related bacteria might lead to a decline in SCFA production, resulting in a higher colonic pH value and increased ammonia production and absorption in the intestine. Thus, *Lachnospiraceae* was also supposed to be a good indicator shown the gut state (36, 38).

We observed that 3% GLC could increase *Bifidobacterium* communities. The *Bifidobacterium* mainly acquires energy from indigestible food debris, especially the complex dietary fiber (39). As reported, the polysaccharides from GL are potential prebiotics, and their supplementation could improve the relative abundance of *Bifidobacterium* (40, 41). According to LDA Effect Size, *Lachnospiraceae*, and *Bifidobacterium* were selected as biomarkers with remarkable differences between treatments, which improved significantly after 3% GLC supplementation. Previous studies have shown that *Lachnospiraceae* and *Bifidobacterium* could promote SCFA production to maintain intestinal homeostasis to a certain degree (37, 42).

The metabolites of intestinal bacteria are relatively small molecules and could intensively influence tissue metabolism. The metabolites could penetrate the gut wall into the blood and are delivered to tissues and organs *via* blood circulation (43). In the current study, the L-beta-aspartyl-L-aspartic acid, nicotinamide riboside, 2-amino-4-[(2-hydroxy-1-oxopropyl) amino] butanoic acid, methionyl-proline, topaquinone, and 8-azaadenosine were selected as biomarkers after screening the differential metabolites by volcanic map analysis.

L-beta-aspartyl-L-aspartic acid or β-aspartylaspartic acid is a sort of dipeptides and are derivatives of asparagine (44). Nicotinamide riboside (NR) serves as a precursor of hepatic nicotinamide adenine dinucleotide phosphate (NADP^+^) and NADPH, which are vital for resistance to oxidative stress (45, 46). The 2-amino-4-[(2-hydroxy-1-oxopropyl) amino] butanoic acid is a sort of alpha amino acids, and have the L-configuration of the alpha-carbon atom. It also known as 2-amino-4-(lactoyl amino) butyric acid, and belongs to organic compounds. To our knowledge, only a few studies have been published on it (HMDB). Methionyl-proline is a dipeptide composed of proline and methionine, which is an incomplete breakdown product of protein catabolism or protein digestion. Some dipeptides are proven to have cell-signaling or physiological effects (HMDB). Topaquinone, also known as the quinone of 2,4,5-trihydroxyphenylalanine, is the cofactor in most copper-containing amine oxidases (47).

The changes in gut microbiota elicited by GLC digestion will inevitably trigger alterations in microbial metabolites. The correlation heat map was established to clarify the potential association between microorganisms and metabolites in broilers treated with 3% GLC. The data showed that the family increased after 3% GLC addition, such as *f__Coriobacteriaceae, f__Synergistaceae*, and *f__norank_o__Oscillospirales* were positively correlated with porric acid A, L-pipecolic acid, and deoxycytidine (Figure 11). The porric acids, is a tricyclic triterpenoid. As a fungal metabolite, it is also found to exhibit antifungal activity (48). The L-Pipecolic acid is a cyclin amino acid. It is derived from L-lysine and an intermediate of the catabolism of D, L-lysine. It participates in various physiological processes in animals, plants, and microorganisms, such as the interactions between organisms, and acts as a precursor of natural bioactive molecules (49).

The *Coriobacteriaceae* family is strictly anaerobic, gram-positive bacteria. The *Coriobacteriaceae* might play an essential role in regulating glucose homeostasis in animals, and it might be beneficial to host glucose metabolism. The previous studies showed that *Coriobacteriaceae* had a strong negative correlation between hepatic glucose and glycogen levels. Moreover, positive correlations were found between *Coriobacteriaceae* and hepatic triglycerides levels in mice and non-high-density lipoprotein cholesterol in the plasma of hamsters (50, 51).

We observed that GLC supplementation could increase the relative abundance of *f-Synergistaceae*. The previous study revealed that most cultured bacteria from the family *Synergistaceae* could ferment amino acids into SCFAs. Certain species might also degrade SCFAs with methanogens *via* syntrophic relationships during anaerobic digestion (52).

The *Oscillospirales*, a butyrate producer (53), is significantly correlated with metabolites, such as LY 235959, 3,6-Ditigloyloxytropan-7-ol, porric acid A, 3,7-dimethyl-5-octene-1,7-diol 1-glucoside, Sumiki's acid, L-pipecolic acid, after 3% GLC supplementation, compared to CON treatment. The *Rikenellaceae*, belonging to the *Bacteroidetes* phylum, might contribute to decreased visceral fat mass. It might act as an adiposity modulator by affecting the production of acetate and propionate (54).

Based on the results above, we proposed that the elevated SCFAs contents in GLC-treated broilers might be positively correlated with the increased SCFAs-related bacteria, such as *Synergistota, Lachnospiraceae, Bifidobacterium, Synergistaceae, Oscillospirales*, and *Rikenellaceae*. Namely, the GLC could influence host health by promoting the relative abundance of SCFAs-related microbe. Despite various links among the metabolism, gut microbiota, and metabolites, this effort only occupied a part portion of the entire metabolic system of the host. Thus, the underlying mechanism of the effects of GLC on broiler's growth and health still needs to be further explored.

## Conclusion

This study determined the effects of GLC supplementation on growth performance, serum biochemical indices, meat quality, intestinal morphology, microbiota, and metabolites. The results revealed that GLC could reduce FCR, increase serum SOD and HDL levels, and enhance cecal SCFA contents. The study also confirmed that GLC could increase bacteria diversity and promote the proliferation of SCFAs-generated bacteria in the gut. Differential metabolites, such as L-beta-aspartyl-L-aspartic acid and nicotinamide riboside, were identified. Overall, GLC could positively affect the health of Sanhuang broilers, and its utilization provides a new target for developing biological feed *via* edible and medicinal fungi fermentation.

## Data availability statement

The data presented in the study are deposited in the Genome Sequence Archive in National Genomics Data Center, accession number CRA010095.

## Ethics statement

The animal study was reviewed and approved by Institutional Animal Care and Use Committee of Guangxi Academy of Agricultural Sciences.

## Author contributions

XL designed the study and analyzed the data. LH, YS, XW, YLuo, SW, YQ, YLu, WZ, and YJ performed the animal experiment and analyzed the tissue samples. YY and YLi revised the manuscript and had primary responsibility for the final content. All authors read and approved the final manuscript.
