# Supplementary material for: Ganoderma lingzhi culture enhance growth performance via improvement of antioxidant activity and gut probiotic proliferation in Sanhuang broilers
Source: Front Vet Sci. 2023 Apr 17;10:1143649. doi: 10.3389/fvets.2023.1143649 (PMC10150954; doi:10.3389/fvets.2023.1143649)
Supplement: Supplementary file 2 [file Table_2.DOCX]

The sequence information is shown in Table S2:

**Table S2** Sequence information

| **Sample Name** | **Valid reads** | **Valid Sequences** | **Average length** | **Total bases** | **Q30** | **Q20** |
| --- | --- | --- | --- | --- | --- | --- |
| M1_1 | 50330 | 38752 | 412.611 | 20766708 | 90.3167 | 96.7676 |
| M1_2 | 48809 | 36833 | 417.314 | 20368696 | 90.492 | 96.8159 |
| M1_3 | 49899 | 37106 | 415.664 | 20741242 | 91.1563 | 97.0875 |
| M1_4 | 46071 | 36811 | 418.503 | 19280869 | 91.3428 | 97.2138 |
| M1_5 | 48543 | 38332 | 416.202 | 20203687 | 90.9538 | 96.9666 |
| M1_6 | 50788 | 36597 | 414.864 | 21070134 | 91.1795 | 97.0643 |
| M2_1 | 45261 | 34703 | 416.554 | 18853630 | 90.3977 | 96.8158 |
| M2_2 | 46356 | 36302 | 415.228 | 19248299 | 90.9202 | 96.9928 |
| M2_3 | 47267 | 35130 | 416.298 | 19677176 | 90.7092 | 96.9151 |
| M2_4 | 50353 | 35423 | 417.025 | 20998451 | 91.4166 | 97.2028 |
| M2_5 | 49946 | 36529 | 416.45 | 20800014 | 91.088 | 96.9956 |
| M2_6 | 50424 | 38170 | 416.446 | 20998877 | 91.6994 | 97.2607 |
| M3_1 | 50882 | 39076 | 415.644 | 21148786 | 90.5051 | 96.8341 |
| M3_2 | 49725 | 34878 | 416.089 | 20690033 | 91.0658 | 97.0441 |
| M3_3 | 48647 | 36534 | 412.783 | 20080667 | 90.5666 | 96.8317 |
| M3_4 | 51680 | 37708 | 414.543 | 21423608 | 91.024 | 97.0538 |
| M3_5 | 48511 | 37996 | 415.482 | 20155449 | 90.7671 | 96.8496 |
| M3_6 | 48091 | 36695 | 414.332 | 19925625 | 91.6519 | 97.2363 |
| M4_1 | 45385 | 35637 | 416.588 | 18906861 | 90.2649 | 96.7478 |
| M4_2 | 50023 | 35343 | 413.839 | 20701492 | 90.7112 | 96.8914 |
| M4_3 | 48721 | 34610 | 417.132 | 20323101 | 90.9235 | 97.0054 |
| M4_4 | 49367 | 36031 | 416.039 | 20538582 | 91.1703 | 97.0641 |
| M4_5 | 47773 | 35140 | 415.163 | 19833591 | 91.1585 | 97.034 |
| M4_6 | 51612 | 38993 | 413.429 | 21337874 | 91.1638 | 97.023 |

Samples: CON, M1_1-M1-6; PCON, M2_1-M2-6; GLC 1, M3_1-M3-6; GLC 2, M4_1-M4-6.
